# Modeling human tumor-immune environments in vivo for the preclinical assessment of immunotherapies

**DOI:** 10.1007/s00262-021-02897-5

**Published:** 2021-04-08

**Authors:** Bethany Bareham, Nikitas Georgakopoulos, Alba Matas-Céspedes, Michelle Curran, Kourosh Saeb-Parsy

**Affiliations:** 1 Department of Surgery, University of Cambridge and NIHR Cambridge Biomedical Campus, Cambridge, UK; 2 Clinical Pharmacology and Safety Sciences, AstraZeneca, BioPharmaceuticals R&D, Cambridge, UK

**Keywords:** Autologous models, Animal models, Immunotherapies, Cancer, Immune-system, Preclinical Safety-assessment/risk management

## Abstract

Despite the significant contributions of immunocompetent mouse models to the development and assessment of cancer immunotherapies, they inadequately represent the genetic and biological complexity of corresponding human cancers. Immunocompromised mice reconstituted with a human immune system (HIS) and engrafted with patient-derived tumor xenografts are a promising novel preclinical model for the study of human tumor-immune interactions. Whilst overcoming limitations of immunocompetent models, HIS-tumor models often rely on reconstitution with allogeneic immune cells, making it difficult to distinguish between anti-tumor and alloantigen responses. Models that comprise of autologous human tumor and human immune cells provide a platform that is more representative of the patient immune-tumor interaction. However, limited access to autologous tissues, short experimental windows, and poor retention of tumor microenvironment and tumor infiltrating lymphocyte components are major challenges affecting the establishment and application of autologous models. This review outlines existing preclinical murine models for the study of immuno-oncology, and highlights innovations that can be applied to improve the feasibility and efficacy of autologous models.

## Introduction

Immuno-oncology (IO) research has shaped our modern understanding of cancer progression, illuminating the paradoxical role of immune cells in both the induction and elimination of cancer. Throughout the stages of tumor development, cancer cells evolve to evade destructive immunity, employing mechanisms that mimic peripheral immune tolerance [1]. Cancer associated inflammation is present at all stages and has been shown to contribute to genomic instability, angiogenesis, epigenetic modifications, induction of cancer cell proliferation and enhancement of anti-apoptotic pathways [2]. Emerging immunotherapies, such as immune checkpoint inhibitors (ICI) (see Glossary) and Adoptive Cell Therapy (ACT), aim to treat cancer through the activation and enhancement of anti-tumor immunity [3]. Immunotherapies have significantly improved overall survival (OS) in a wide variety of cancer types, yet immunotherapeutic agents have a notably higher rate of failed clinical trials in comparison to alternative cancer therapeutics [4]. Preclinical assessment of the safety and toxicity of immunotherapies is heavily reliant on in vivo immunocompetent mouse models that show poor translation of beneficial responses to the clinic. Furthermore, the complex heterogeneity of immune systems, both between and within patients over time, significantly impacts the effectiveness and safety of approved immunotherapeutic agents [5]. Immunotherapies have been linked to numerous immune related adverse events (IRAEs) such as cytokine release syndrome (CRS), pneumonitis, and neuropathy, in addition to late onset rheumatic diseases [6–9]. These IRAEs range from mild to severe and are thought to be linked to inflammatory damage consequent to non-specific activation of the immune system. There is a clear need for well-designed in vitro and in vivo preclinical platforms that can accurately assess both the efficacy and safety of novel agents and individual responses to immunotherapies.

Human immune system (HIS) mouse models are increasingly being combined with human xenograft models for the investigation of human immune cell behavior in response to tumors and therapeutics [10, 11]. Most notably, HIS patient-derived xenograft (hu-PDX) models have been utilized to assess efficacy and toxicity of immunotherapies in the treatment of a variety of tumor types [12, 13]. However, the rate of successful engraftment is highly variable and it can take at least 3 months to develop an experimental cohort [14]. Patient-derived organoids (PDOs) are emerging as suitable alternatives, retaining the complexity of the original tumor, and showing quicker and more successful establishment than traditional PDXs [15, 16]. Furthermore, preclinical HIS-tumor models often rely on allogeneic lymphocytes from donated fetal liver (FL), bone marrow, cord blood or peripheral blood mononuclear cells (PBMCs) for immune reconstitution [10]. This allogeneic reconstitution is not representative of the clinical scenario and can lead to difficulties distinguishing between anti-tumor and alloimmune responses.

Models that integrate autologous patient-derived tumor and immune cells can provide a more individualized platform for IO research. Emerging autologous HIS models have shown promise as a valuable tool for the mechanistic and preclinical study of approved and novel immunotherapies [17–21]. The use of autologous models is currently hindered by four key challenges: access to autologous tissues, immune reconstitution in vivo, Modeling of the entire tumor microenvironment (TME) and Modeling longitudinal therapeutic responses. This review will outline existing and emerging in vivo platforms for the preclinical testing of immunotherapies. Moreover, the key challenges to establishing autologous models will be addressed, with potential solutions offered to increase their feasibility and applicability.

## Murine IO models

### Immunocompetent mouse models

**Fig. 1:**
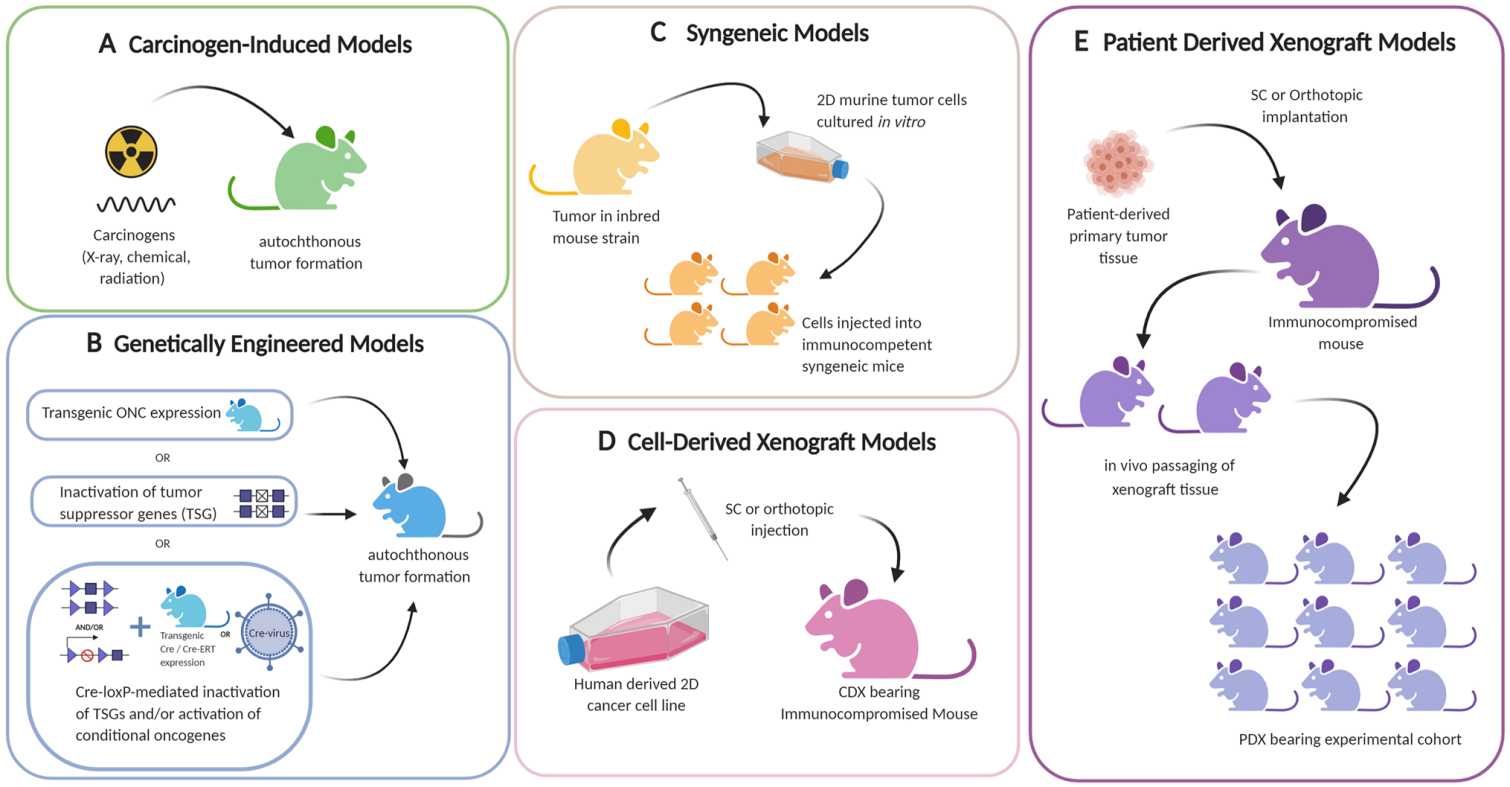
Schematic showing the generation of common preclinical murine cancer models. **a.** Carcinogen-Induced models are generated through the administration of cancer-causing agents to immunocompetent mouse strains. **b.** GEMMs promote tumor development through the promotion of oncogene expression or through deletion of tumor suppressors. **c.** Syngeneic models are generated through administration of murine-derived tumor cell lines, commonly carcinogen-induced cancers or transgenic tumor lines. **d.** CDX models are established through transplantation of human-derived 2D cancer cells into immunocompromised mice, either subcutaneously (under the skin) or orthotopically (in the corresponding organ). **e.** PDX models are established by transplantation of whole patient-derived tumor tissue either subcutaneously or orthotopically into immunocompromised mice, followed by in vivo passage of the tissue to create an experimental cohort

Preclinical study of immune-tumor interactions is largely dominated by immunocompetent murine models in the form of carcinogen-induced, syngeneic, or genetically engineered mouse models (GEMMs) (Fig. 1, Table 1). Carcinogen-induced mouse models are established through the administration of cancer-causing agents, such as chemicals and biological toxins, and best reflect spontaneous tumor formation for the study of therapeutic effects of tumor initiation, promotion, and progression [22, 23]. Carcinogenesis models have contributed greatly to our understanding of immune surveillance and mechanisms of immune editing [24]. However, the use of carcinogens to promote cancer development can lead to severe DNA damage, resulting in the accumulation of somatic mutations. Furthermore, most human cancers do not arise in response to carcinogen exposure alone [25]. Autochthonous tumor formation can also be modeled with GEMMs through either promotion of oncogene expression or deletion of tumor suppressors [26]. GEMMs allow for the Modeling of tumor tissue in both a cancerous and pre-cancerous form and can provide great insights into the immunobiology of cancers. However, the spontaneous nature of tumor formation seen in both carcinogen-induced and GEMM models can be difficult to monitor and require large cohorts to produce meaningful data due to the high variability in tumor formation and progression [22]. For example, the p53^−/−^ mouse can take more than 7 months to form primary tumors and show morbidities soon after formation, limiting opportunities for the evaluation of novel therapeutics [27]. Furthermore, GEMMs do not recapitulate the entire multistep tumorigenesis seen in humans [28], often having only two driver mutations [25].

**Table 1:** In vivo preclinical models for Immunotherapy

Model	Advantages	Limitations	References
*Immunocompetent models*			
Carcinogen-induced models: Tumor bearing mice induced after administration of carcinogen	+ Sporadic cancer development + High heterogeneity + Natural tumor microenvironment + Tumors develop from normal cells + Easy to work with + A wide range of methodologies can be incorporated to induce tumors	‒ Difficult to monitor tumor growth ‒ Variability in tumor progression and high heterogeneity ‒ Large cohorts needed for data interpretation ‒ Do not mimic tumor formation from chronic inflammation ‒ Severe DNA damage ‒ Limited human cancers purely derived from carcinogen exposure	[22–25, 31]
GEMMs Tumor bearing mice established through genetic manipulation of cancer causing genes	+ Natural tumor microenvironment + Tumors development from normal cells + Modeling cancer at a variety of stages	‒ Low immunogenicity ‒ Difficult to monitor tumor growth ‒ Lengthy and variable tumor progression ‒ Costly and challenging breeding and gene manipulation process ‒ Genomic homogeneity	[22, 25- 27, 31]
Syngeneic Models Tumor bearing mice established through injection of murine cancer cell lines	+ Reproducible + Easy establishment of large cohorts + Accurate tumor monitoring + Non-immunogenic + Low cost	‒ Lack of native tumor microenvironment ‒ Methodology linked alteration of immunophenotype ‒ Lack heterogeneity ‒ Limited host strains	[26, 29–31]
*Humanized Models*			
Hu-CDX Immunocompromised mice bearing human tumor cell line xenograft and reconstituted with a HIS	+ High engraftment rates and reproducibility + Inexhaustible tumor source + Potential for metastasis when transplanted orthotopically + CDX models of a variety of tumor types readily available commercially	‒ Highly selective in vitro expansion resulting in genetic and phenotypic aberrations ‒ Low predictive power and correlation to clinical results ‒ Limited by the simplicity of 2D cell cultures	[13, 25, 46, 52–54]
Hu-PDX Immunocompromised mice bearing whole tissue human tumor xenografts and reconstituted with a HIS	+ Retention of tumor cell heterogeneity and stromal tissue (at low passages) + Reproduces the complexity of the original tumor and immune system + Have been established for a wide variety of tumor types, including drug refractory tumors + Allogeneic models readily available commercially	‒ Low tumor engraftment success rate (approximately 49%) ‒ Engraftment favors aggressive tumors ‒ Long establishment times (at least 3 months) ‒ Low rates and duration of immune reconstitution (dependant on humanization method) ‒ Onset of GvHD shortening experimental window ‒ Costly	[10, 14, 44]
Hu-CAR Immunocompetent mice bearing human Tumor xenograft and HIS and administered a CAR therapeutic	+ Recapitulate post treatment immune changes + Measure CAR mediated killing (both direct and indirect via activation of resident immune cells) + Facilitates the design of new CAR therapeutics	‒ Limited IL-6 expression in these models ‒ Rapid onset of GvHD ‒ Not able to model resistance over time	[17, 18, 63]

In contrast, in syngeneic models, wild type mice are transplanted with spontaneous, carcinogen-induced, or transgenic tumor cells established in syngeneic inbred mouse strains, allowing for low-cost establishment of large experimental cohorts [26, 29]. The lack of rejection in these models also allows for the longitudinal study of tumor survival, growth, and metastasis. Despite these strengths, choice of syngeneic strain and tumor induction methodology has been shown to alter tumor immunophenotypes, treatment response and mutational burden within the same cancer types [30]. Inconsistencies between these models can make it difficult to distinguish clinically relevant data from technique-related artifacts, hindering the development and validation of novel therapeutics. The utility of immunocompetent mouse models is further diminished by their dependence on murine tumor tissue and murine immune compartments. Beneficial responses seen in preclinical murine models have thus rarely translated to a clinical setting. This is likely to be due to the differences between human and mice tumor biology, as well as the alignment of protein expression and signaling of their respective immune systems [26, 31]. Reconstitution of immunocompromised mice with a HIS is therefore increasingly being used as an alternative tool for the study of human immune tumor interactions.

### Human immune reconstitution

**Fig. 2:**
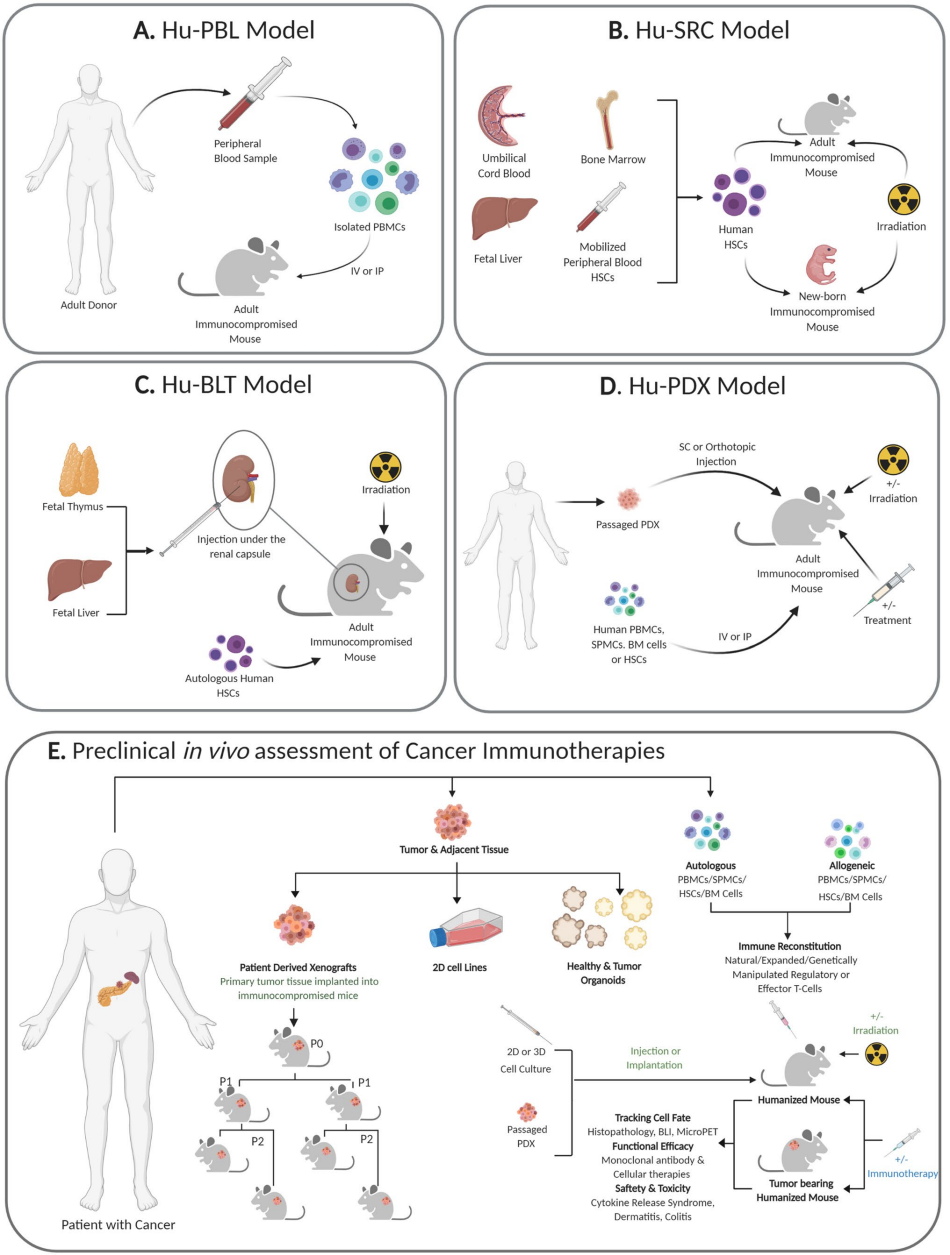
Steps involved in establishing human immune system (HIS) models *in vivo*: **a**. The hu-PBL (human Peripheral Blood Leukocytes) model can be established through intravenous (IV) or Intraperitoneal (IP) injection of human peripheral blood mononuclear cells (PBMCs) in adult immunocompromised mice. **b**. The hu-SRC (human Stem Repopulating Cell) model is established through either IV or intrafemoral (IF) injection of haematopoietic stem cells (HSCs) into irradiated adult immunocompromised mice. HSCs are isolated from either umbilical cord blood, bone marrow (BM), fetal liver or mobilized peripheral blood HSCs. Hu-SRC models can also be established through IV, intracardiac (IC) or intrahepatic (IH) injection of HSCs into irradiated newborn immunocompromised mice. **c**. The hu-BLT (human Bone marrow, Liver, Thymus) model can be established through the transplantation of foetal thymus and liver fragments under the kidney capsule of irradiated adult immunocompromised mice, in addition to the IV injection of autologous HSCs. **d**. The hu-PDX (HIS patient-derived xenograft) model dually engrafts immunocompromised adult mice with early passage PDXs and a HIS. Most commonly, this is done with the use of immunocompromised mice engrafted with human CD34^+ve^ HSCs but can also be established with human lymphocytes such as PBMC or splenic mononuclear cells (SPMCs). **e**. Schematic showing the development of preclinical models for the *in vivo* assessment of cancer immunotherapies. Primary tumor tissue from the patient can be used to derive 2D cell lines and PDOs. A biopsy of primary tissue can also be expanded *in vivo *in immunocompromised mice to establish a PDX line. Patient derived (autologous) or allogeneic PBMCs, SPMCs or HSCs can be used to generate humanized mice. 2D cells, 3D cells or passaged xenograft tissue can be implanted or injected into the established HIS mice. Both HIS mice and tumor bearing HIS mice can be used to study cell fate, functional efficacy and safety and toxicity of immunotherapies.

HIS models reconstitute immunocompromised mice, most commonly NOD.*Cg-Prkdc*^scid^ *Il2rg*^tm1Wjl^/SzJ (NSG) strains [32], with a human-derived immune compartment. There are currently three established HIS platforms; human Peripheral Blood Lymphocytes (hu-PBL), human Stem Repopulating Cell (hu-SRC) and human Bone marrow, Liver Thymus (hu-BLT) model (Fig. 2). Curran et al. review outlines these platforms, their pathology and application to the field of IO [10]. In short, in the hu-SRC model, mice are humanized through the engraftment of CD34^+ve^ hemopoietic stem cells (HSCs) obtained from bone marrow (BM) [33], umbilical cord blood (UCB) [34] or FL [35]. This model leads to engraftment of a diverse repertoire of immune cells, including B- and T-cells, with improved reconstitution when using UCB or FL derived HSCs in newborn rather than adult mice [36]. However, this model typically takes 16 weeks to be established, displays a lack of human educated T-cells and is heavily reliant on pre-experimental sub-lethal γ-irradiation to facilitate engraftment [37]. In comparison hu-BLT models are established through the surgical transplantation of human FL and thymus fragments under the kidney capsule of immunocompromised mice. The presence of autologous thymic tissue allows for better T-cell development and education, in addition to supporting a more diverse immune lineages of B-cells, monocytes, macrophages and dendritic cells [38, 39]. A consequence of the T-cells maturing in the fetal thymus is the development of graft *versus* host disease (GvHD), in which maturing T-cells attack the mouse tissue, shortening the experimental window to approximately 20 weeks [40]. This immune attack on the mouse is avoided in the hu-SRC model, as human T-cells are educated by the mouse thymus with negative selection of T-cells most likely to attack mouse antigens [41]. However, this education of human T-cells against antigen presented by mouse thymic epithelial cells can make the hu-SRC model unsuitable for the study of immunotherapies with T-cells largely unable to recognize and bind to potential human antigen present by human (rather than mouse) antigen presenting cells [42].

The hu-PBL models are the simplest and most cost-efficient method of humanization, engrafting immunocompromised mice with mature human leukocytes isolated from peripheral blood, spleen or lymph nodes. As human leukocytes inoculated in this model are already mature, humanized mice can be established quickly with a predominant reconstitution of CD3^+ve^ T-cells with an activated/effector phenotype [10]. The engraftment kinetics of the hu-PBL model make it best suited for the study of mature immunity, such as identification of T-cell clones responsive to tumor neoantigens [43]. The hu-PBL model can also produce more appropriate tumor-immune interactions, as T-cells educated within a human environment are more likely to recognize and bind to MHC proteins on the implanted human tumor [44]. However, the presence of mature T-cells significantly shortens the experimental window with this model, rapidly inducing GvHD [45]. Despite these limitations, hu-PBL models are the most feasible humanization method for establishing autologous HIS-tumor models for adult patients and show promise as a predictive model of patient response rates [43].

#### Humanized CDX models

Cell line-derived xenograft (CDX) models establish a tumor bearing system in vivo through implantation of immunocompromised mice with cell lines derived from primary tumor tissue. CDX bearing HIS mice (hu-CDX) have been utilized to investigate tumor-immune compartment interactions, as well as for the generation of novel cancer immunotherapies [46]. Rios-Doria et al.utilized CD34^+ve^ humanized NSG mice transplanted with different cancer lines to demonstrate that TIL populations were influenced by tumor type and not by donor. Furthermore, the study demonstrated that cancer cell lines expressed programmed death ligand 1 (PD-L1), while they showed efficacy of the anti PD-L1 therapeutic agent, atezolizumab, in their hu-CDX model [13]. Other studies have utilized hu-CDX mice to interrogate efficacy of bi-specific antibodies for targeting T-cells to a tumor antigen [47–49] or use of adoptive natural killer (NK) cell therapy either alone or in combination with therapeutic antibodies to target tumors [50, 51].

Hu-CDX models are an attractive preclinical tool as the xenotransplantation of human cell lines is easy to control, well established and allows for rapid evaluation of therapeutic agents. However, preclinical studies using hu-CDXs are not able to predict treatment safety and efficacy in humans, with failures in clinical trials linked to the genetic homogeneity of the two-dimensional (2D) cell lines used in these models [25]. 2D cells implanted in vivo often do not recapitulate the biological properties and behavior of the original tumor, with differences in metastasis, TME and immune infiltration [52, 53]. The unrepresentative nature of cell line xenografts was highlighted in Daniel et al.*’*s CDX model of small cell lung cancer (SCLC). In this study, tumor-specific genes expressed in primary SCLC tissue were lost throughout cell culture and not regained in vivo*,* with significant implications for preclinical drug screening using these models [54]. The lack of translational results seen in hu-CDXs has driven the adoption of models that can better retain the complexity of the parental tumor, such as patient derived xenografts (PDXs).

### Humanized PDX models

PDXs are established from surgically dissected tumor fragments directly engrafted, subcutaneously or orthotopically, into immunocompromised mice (Fig. 2). Compared to 2D tumor cell lines, these models better reproduce the complexity of human cancer, preserving biological and histopathological features of the original tumors [14]. PDX-bearing HIS mice (hu-PDX) have emerged as a platform to evaluate the efficacy of immunotherapies in a range of tumor types. For example, human anti-carbonic anhydrase IX (anti-CAIX) antibodies have been shown to promote immune-mediated tumor killing in a PBMC humanized hu-PDX model of renal cell carcinoma (RCC) [55]. Combination therapies have also been evaluated in hu-PDXs, such as CTLA-4 blockade and the oncolytic vaccinia virus (VACV) therapy [56]. PDXs can survive in vivo with an allogeneic immune compartment, however immune reconstitution with different donors has been shown to alter treatment response in the same PDX tumor bearing mice [57]. Ideally, hu-PDX models should be generated with the HIS and tumor derived from the same donor, allowing the assessment of therapeutics in the context of the patient’s own immune system. However, poor tumor implantation success rates and limited access to patient immune samples are two major limitations of this approach (Table 1) that often make autologous reconstitution unfeasible.

Consequently, the tumor and immune cells used in this model are usually obtained from different, or partially human leukocyte antigen (HLA) matched, donors. Although these studies can be very informative, variations in the anti-tumor immune response from different donors to the same PDX tumor, due to an underlying allogeneic response, can complicate data interpretation [57]. Recent studies have utilized tumor infiltrating lymphocytes (TILs) as an autologous source for immune reconstitution, developing autologous hu-PDX models for the study of immunotherapies [20, 21]. For example, Jespersen et al.modeled ACT and anti-PD1 immunotherapy in hu-PDX mice reconstituted with autologous TIL-derived T-cells and reported response rates that correlated with clinical outcomes. This study also highlighted the need for repeated administration of IL-2 for T-cells to effect tumor eradication, even in the presence of PD-1 antibodies [20]. Hu-PDX models have also been utilized in the field of Allergo-Oncology, which is concerned with immune and allergic responses in cancer [58]. For example, in a hu-PDX model of ovarian cancer, IgE antibodies were used to prime tumor associated macrophages (TAMs) to elicit effector responses and restrict tumor proliferation [59, 60]. Whilst further comparisons of hu-PDX models is warranted, emerging autologous hu-PDXs show the value of autologous reconstitution in preclinical models, recreating the anti-tumor effects observed in patients.

### Humanized CAR models

Chimeric antigen receptor (CAR) T-cell therapy aims to activate anti-tumor immunity through reprogramming a patient's immune effector cells to recognize tumor surface antigens. Patient-derived T-cells are transduced, often through lentiviral vectors, with a construct containing a single chain variable fragment (scFv), transmembrane domain, co-stimulatory molecule and stimulatory molecule [61]. Two CAR T-cell therapies have been approved for clinical use (Kymriah™ and Yescarta™), both of which target CD19-expressing tumor cells for use in acute lymphoblastic leukemia and B-cell Lymphomas [62]. Both Kymriah™ and Yascarta™ have shown significant lifesaving potential in otherwise incurable cases, although patients must be closely monitored for severe treatment linked toxicities such as CRS, neurotoxicity (NT) and tumor lysis syndrome (TLS) [63]. Furthermore, resistance often occurs in these patients only months after treatment [63–65]. Therefore, further research is essential to understand the mechanisms of treatment resistance and toxicities, and to develop novel CAR T-cell therapies. Emerging autologous HIS models are a highly valuable tool for mechanistic and preclinical study of CAR T-cell therapies, avoiding anti-mouse xenoantigens and allogeneic responses [17]. For example, Jin et al. recently developed an autologous HIS mouse model of anti-CD19 CAR-T cell therapy, in which immunocompromised NSG mice were reconstituted with a functional human immune system that was genetically matched to B-lymphoblastic leukemia (B-ALL) [18]

Whilst CAR-T cell therapies have shown great promise for treating hematological cancers, they are limited in their ability to reach solid tumors due to a broad array of immune obstacles. TAMs naturally traffic to tumors and may be able to overcome the barriers CAR-T cells face. Novel CAR-Macrophages (CAR-Ms) are being developed and modeled in HIS mice for the treatment of solid tumors. Klichinsky et al. tested anti-tumor activity of a CAR-M to implanted SKOV3 tumor cells in CD34^+ve^ HSC humanized NSG mice [64]. In this model, CAR-Ms from a male donor and HSCs from a female donor were used to correctly distinguish HSC and CAR derived Macrophages. CAR-Ms were shown to boost anti-tumor T-cell activity and illicit a pro-inflammatory response within the TME through expression of M1 associated interferon genes [64]. Humanized models can thus enable preclinical validation of CAR therapeutics. However, studying therapy toxicity in HIS models is still limited due to undetectable IL-6 levels in mice, with IL-6 being a known driving force for CRS [17]. Furthermore, the onset of GvHD limits the evaluation of treatment resistance over time. Since both toxicity and relapse are of great concern when choosing a treatment plan, there remains an urgent unmet need for preclinical models that can more accurately recapitulate the clinical response.

## Autologous IO models: limitations and innovations

Preclinical models utilizing patient derived tissues are essential to the advancement of cancer therapeutics, with anticipated improved predictive power than classical animal models. Autologous models, using tumor and immune compartments from the same patient, have great potential to better represent patient immune-tumor interactions and treatment responses. However, several confounding factors hinder the establishment of autologous models, making them unfeasible for many researchers. These include poor access to autologous tissues, donor- and technique-related variation in immune reconstitution, poor representation of the in vivo TME and limited ability to model longitudinal responses due to a short experimental window. It is important to improve access and application of autologous models to truly unveil their potential as a preclinical tool. We outline these limitations below and review methodologies that could potentially address these challenges (Table 2).

**Table 2:** Overcoming limitations of preclinical autologous models

Limitation	Potential solution	Future work	References
Access to autologous Lymphocytes and tumor cells	PBMC and TIL expansion protocols Use of alternative lymphocyte sources such as SPMCs 3D tumor organoids can be used as a substitute when tumor tissue is limited	Optimization of expansion protocols to allow for a greater lineage of lymphocyte populations Assessment of humanized PDO (hu-PDO) mouse models for the Preclinical study of immunotherapies	[10, 15, 16, 20, 21, 77]
Limited immune reconstitution	Preliminary colony-forming assay to assess repopulation capacity Use of patient bone marrow cells when possible Use of novel strains with transgenic expression of human Molecules that promote engraftment of human immune Compartments	Investigate and clarify benefits of novel strains in hu-PBL models Investigate and clarify disparities in tumor-immune interactions When using adult BM cells for immune reconstitution Explore methods of improving engraftment success for donors with low repopulation capacity	[10, 19, 81, 82]
Modeling the parental TME	Expanding PDXs orthotopically in already humanized mice Injection of PBMC derived MSCs to promote neovascularization Complimentary use organoid wholistic (ALI), reductionist (tumor-stromal/immune) and microfluidic (‘tumor-on-chip’) coculture systems	Further exploration of the implantation of tumor and stromal PDOs and PDO co-cultures in vivo	[44, 85–91]
Rapid onset of GvHD	Novel strains of immunocompromised mice with MHC class I and or II knockout (such as the NSG-dKO strain) can be used to lengthen experimental windows	More research needed using MHC knockout strains in preclinical study of immunotherapies Exploration of potential cross strains that can both improve immune engraftment and prolong the experimental window	[77, 92–95]

### Access to autologous tumor tissue

Matched patient tumor and immune cells is often a scarce resource and many researchers rely on archived tissue, immortalized cell lines and allogeneic immune cells for their studies. Primary tumor tissue and adjacent healthy tissue, although routinely removed during surgical treatment of cancer, is usually rapidly formalin-fixed-paraffin-embedded (FFPE) for invaluable diagnostic purposes. Acquiring fresh tissue samples requires a high level of collaboration to collect limited samples in a timely manner, so as not to adversely impact the patient’s treatment or diagnosis. It is essential that researchers can utilize the limited supply of primary tissue to establish representative preclinical models. Whilst 2D cell cultures can provide an unlimited supply of tumor cells, they do not recapitulate the complexity of the original tumor [66]. Furthermore, traditional PDX models can take up a considerable amount of primary tissue to establish, with unpredictable rates of successful engraftment [14].

Alternatively, three dimensional (3D) cell cultures, such as organoids, can be established in a wide range of cancer types and have been shown to more closely mimic the in vivo tumor biology in terms of histoarchitecture [16, 67], mutational landscape [68, 69], signaling [70] and heterogeneity [71, 72]. This phenotypic complexity allows for physiological barriers to immune cells and increased resistance to cytotoxicity, more closely replicating in vivo tumor-immune interactions [73]. Tumor PDOs can be established from a small tumor biopsy and have been shown to have a higher rate of successful establishment in comparison to PDXs in addition to retaining the genetic and phenotypic complexity of the parental tumor [15, 16]. Unlike PDXs, organoids can be established from both tumor and adjacent healthy tissue, providing autologous healthy organoids that can serve as experimental controls. Furthermore, PDOs can be easily cryopreserved and utilized for in vitro characterization and experimentation, allowing for more freedom in experimental design.

### Access to autologous immune compartments

PBMCs and TILs are often the only fresh source of autologous lymphocytes for preclinical models and are ideal for the establishment of in vitro co-culture assays, yet provide an insufficient cell yield for in vivo experimentation. Due to donor variation in the success of human immune engraftment, multiple in vivo reconstitutions may be required to optimize humanization, therefore a plentiful supply of immune cells is essential. Ex vivo culture and expansion of patient lymphocytes could be used to address this limitation. Several expansion protocols are available and can be optimized for individual lymphocyte and monocyte populations, maintaining relative phenotypes [65, 74–76]. For example, Issa et al.published a protocol for the ex vivo expansion, characterization, and in vivo implantation of human regulatory T-cells [75]. As mentioned, ex vivo expansion of TILs has also been utilized for the establishment of autologous hu-PDX models [20, 21]. Gitto et al. achieved an 8–tenfold expansion of TILs after 12–14 days consisting of 90% CD3^+ve^ T-cells, 71.2% of which were cytotoxic CD8^+ve^ T-cells [21]. Whilst not representative of the entire immune compartment, expansion protocols could allow for more in-depth exploration of interactions between tumor cells and specific immune populations.

In some cancers, additional sources of autologous lymphocytes will be available. For example, BM cells may be available in patients undergoing BM aspiration and/or biopsy as part of their treatment. Furthermore, spleen mononuclear cells (SPMCs) can be obtained from pancreatic cancer patients undergoing surgical resection with splenectomy. The spleen is a plentiful resource of autologous mature lymphocytes, and SPMCs could be used for humanization of immunocompromised mice, as in the hu-PBL model [10]**.** Recently Matas‐Céspedes et al. assessed the efficacy of SPMCs for establishing HIS mice, comparing BM, HSC, PBMC and SPMC engraftment from the same human donors. They demonstrated that SPMCs can be used for the successful engraftment of immune cells, showing hCD45 + ve cells in the circulating blood, spleen and liver of the mice. Similar to the hu-PBL model, engrafted immune cells from SPMC reconstitution was mostly T-cells with an effector memory phenotype [77].

### Improving immune reconstitution

The hu-PBL model, whilst simple and cost effective, is limited to the engraftment of mature T-cells and low levels of B-cells and myeloid cells [78]. Reconstitution of immunocompromised mice with BM derived CD34^+ve^ cells has shown systemic repopulation of multiple lineages and presents a suitable alternative to the hu-PBL model in patients undergoing fibular resection and BM aspiration. For example, Fu et al.'s model of head and neck squamous cell carcinoma (HNSCC) transplanted autologous HNSCC tumor cells and CD34^+ve^ BM cells from HLA-A2^+ve^ patients into NOG-A2^+ve^ mice. In this model, both human lymphoid and myeloid cells were shown to infiltrate the TME and appropriate populations of human T-cells, myeloid cells and NK cells were identified in the spleen of the mouse [19]. However, the use of CD34^+ve^ cells from adult donors has several limitations when compared to CD34^+ve^ cells derived from umbilical cord blood, including lower pluripotency, quiescence and lymphopoiesis in addition to poorer engraftment efficacy [79, 80]. To improve reconstitution a preliminary colony-forming unit assay could be used in vitro to discriminate donors with higher repopulation capacity, which can then be selected for in vivo engraftment of mice. Furthermore, variation between experiments can be reduced by selecting donors within the same age group, preferable young adults (> 35 years old) but mid-age adults (36–61) can also be considered [80].

Strains with transgenic expression of human cytokines can also be employed to improve immune reconstitution through cytokine stimulation of immune cell hematopoiesis [10]. For example, NSG-SGM3 mice express human cytokines; SCF, GM-CSF and IL3, and have been shown to support myeloid and CD4^+ve^ Treg engraftment in hu-SRC models [81]. NSG-IL15 mice were also shown to promote NK survival post HSC engraftment in comparison to NSG mice [82]. However further research is required to clarify the benefits of these strains in hu-PBL and hu-BLT models.

### Modeling the tumor microenvironment

The TME is comprised of a wide range of resident and infiltrating host cells, secreted factors, and extracellular matrix proteins in addition to heterogenous tumor cells. Each component of the TME is thought to play a role in tumor formation and survival. For example, cancer-associated fibroblasts (CAFs) have a well-established role in tumor promotion primarily through increasing vascularization and blood flow to the TME [83]. Models that fail to account for all components of the TME cannot presume to be representative of the parental in vivo environment. However, Modeling the TME in its entirety remains challenging, with most autologous models comprising of immune and tumor cells only. Human stromal cells present within PDXs are replaced with mouse stroma after approximately 3 passages [84]. Retention of this parental stromal tissue can be improved by expanding PDXs in already humanized mice, allowing for regulation of xenograft growth by the human immune system [44]. Neovascularization could also be promoted in hu-PDX models through additional inoculation with peripheral blood derived mesenchymal stem cells (MSCs), previously shown to upregulate vascular endothelial growth factor (VEGF) in NSG mice [85]. The TME can further recapitulated through orthotopic transplantation of tumor tissues, which has been shown to better retain the parental morphology, molecular and pharmacological features in comparison to subcutaneous transplantation [86].

Novel in vitro co-culture systems could also be used to compliment in vivo models and provide important insights into TME biology. PDOs can be easily co-cultured with a variety of cell types and can be used to provide insights into the role of specific TME compartments, such as CAFs [87]. Furthermore, holistic co-cultures can be achieved with the use of more complex culturing systems such as air liquid interfaces (ALIs). For example, Neal et al. cultured tumor biopsies in an ALI, preserving the endogenous immune cell population and T-cell receptor (TCR) spectrum for up to 30 days. Their culture system also allowed Modeling of immune checkpoint blockade with subsequent T cell expansion and tumor killing [88]. Microfluidic models, such as ‘tumor-on-chip’ 3D culturing systems, have also emerged to mimic the dynamic in vivo environment more closely. In this approach, tumor spheroids are placed within a microfluidic device and continuously supplied nutrients and oxygen in addition to waste removal [89, 90]. These systems allow for study of more complex aspects of the TME, such as chemical gradients [91].

### Modeling longitudinal immunotherapy responses

The main disadvantage of hu-PBL models is the rapid onset of GvHD, limiting the experimental window to short term studies. Whilst the hu-SRC model has a lower incidence of GvHD these models are not efficient for autologous studies due to their use of HSCs [10]. Novel strains of immunocompromised mice have been developed to delay GvHD through elimination of major histocompatibility complex (MHC) class I and/or II expression [92–94]. Most recently, the use of NSG-(*K*^*b*^* D*^b^)^null^(*IA*)^null^ (NSG-dKO) mice, deficient in murine MHC I and II, was shown to significantly delay GvHD post PBMC engraftment (up to 125 days) [93] and SPMC engraftment (up to 140 days) [77]. This improved survival is consistent with xenoreactivity being predominantly driven by murine MHC class I and II molecules. These emerging strains provide a platform for longitudinal evaluation of immunotherapies; however, reconstitution is still limited to CD8^+ve^ and CD4^+ve^ mature T-cells. Crossing NSG MHC KO strains with other strains such as NSG-SGM3, may both delay GvHD onset and support the expression of human cytokines [95], providing a slightly more representative in vivo environment.

## Conclusions

HIS mouse models, combined with human tumor xenografts, are rapidly becoming the gold standard for the preclinical assessment of immunotherapeutic agents. The method of humanization has significant impacts on the resulting in vivo immune repertoire, T-cell functionality and experimental window. Therefore, researchers must choose models that best align with their research questions. For the establishment of mice with autologous human immune cells and tumor cells, the hu-PBL appears to be the most feasible and reliable choice. Humanized xenograft models, such as hu-PDX and hu-CDX, utilize immunocompromised strains to combine human tumor and immune compartments. Although hu-CDX models are attractive for rapid high-throughput screening, hu-PDX better retain the complexity of human cancer showing better translation of results. The key confounding factor in these models is the use of allogeneic tumor and immune cells, thus results could be misinterpreted due to the presence of alloantigen responses.

Models that comprise of an autologous patient derived-tumor tissue and immune compartment provide a more representative system to study tumor-immune interactions and patient-specific responses. However, several challenges reduce the potential of autologous models. In this review, we have outlined how access to autologous tissues can be improved with the use of hu-PDOs to retain the complexity of the parental tumor and improve upon the poor engraftment success and long expansion times of traditional PDXs. Furthermore, resources of autologous immune compartments can be improved with the use of expansion protocols and the use of SPMCs or BM cells in applicable cases. The engraftment success of these models can be improved with preliminary screening for engraftment efficacy and the employment of transgenic strains expressing human cytokines. Transgenic strains can also be utilized to improve the experimental window of these models. Finally, the TME can be better represented in these models through the alteration of PDX expansion and transplantation methodologies, the addition of MSCs and the utilization of complimentary in vitro 3D co-cultures. Whilst there is no current perfect solution, we expect to witness a surge of autologous models in the coming years with the fine tuning of these in vitro and in vivo methodologies.

## Glossary

**Adoptive Cell Therapy (ACT)**Form of immunotherapy that aims to reprogram a patient’s immune system, typically T-cells, to recognize and destroy cancer.

**Air Liquid Interface (ALI)**A method of cell culture in which basal stem cells are grown with basal surfaces emerged in media, and the apical cellular layer exposed to air.

**Allogeneic**Cells taken from different individuals of the same species.

**Autologous**Cells taken from the same individual.

**Cancer-Associated Fibroblast (CAF)**One of the most abundant components in the TME.

**Chimeric antigen receptor (CAR)**A receptor that has been designed to bind to tumor specific proteins. CARs are added to immune cells, typically patient derived T-cells, to help T-cells find and kill tumor cells.

**Cell Derived Xenograft (CDX)** models inject cell lines derived from patient tumor into a mouse.

**Cytokine Release Syndrome (CRS)**A condition where there is a large and rapid release of cytokines into the blood as a result of immunotherapy treatment. Symptoms of CRS vary in severity from a mild reaction to severe or life threatening.

**Immune Checkpoint Inhibitors (ICI)**Monoclonal antibodies that target immune checkpoints to prevent tumor immunogenicity. Common targets of ICIs are CTLA-4, PD-1 and PD-L1.

**Graft vs Host Disease (GvHD)**A post-transplantation condition that occurs when donated tissues or cells see the healthy tissues in the recipient’s body as foreign and attack them. Typical GvHD symptoms in mice are weight loss, hunched posture, and reduced mobility. GvHD can be fatal in mice and so when these symptoms reach a certain severity the mouse is culled.

**Major Histocompatibility Complex (MHC)**A group of genes that code for proteins found on the surface of cells, MHC molecules bind peptide fragments from pathogens and display them on the cell surface for T-cell recognition. MHC molecules can be divided into class I (found on almost all cells) and class II (restricted to macrophages and lymphocytes).

**NOD.Cg-Prkdc**^**scid**^** Il2rg**^**tm1Wjl**^** /SzJ (NSG)**Strain of immunocompromised mice, developed by the Jackson Laboratory, that lack T-cells, B-cells and NK cells. NSG mice carry mutations on the NOD/ShiLtJ genetic background, severe combined immune deficiency (*scid*) mutation in the *Prkdc* protein and a complete null allele of the IL2 receptor common gamma chain.

**Programmed cell death protein 1 (PD-1)**A immune checkpoint protein that, alongside its ligand, plays an essential role in the inhibition of T-cells within the tumor microenvironment.

**Patient Derived Organoid (PDO)** Organoids derived from a patient tumor for in vitro analysis or for transplantation into a mouse.

**Patient Derived Xenograft (PDX)** models implant small samples of primary human tumor tissue and into an immunodeficient mice for research purposes.

**T-Cell Receptor (TCR)**A protein complex found on the surface of T-cells that is responsible for the recognition of antigen-major histocompatibility complex, and initiation of inflammatory responses.

**Tumor Microenvironment (TME)**Includes heterogenous tumor cells in addition to resident and infiltrating host cells, blood vessels, secreted factors and extracellular matrix proteins within and surrounding the tumor site.

## Abbreviations

ACT: Adoptive cell therapy
ALI: Air liquid interface
BM: Bone marrow
CAR: Chimeric antigen receptor
CAF: Cancer-associated fibroblasts
CAR-M: CAR-macrophages
CDX: Cell derived xenograft
CRS: Cytokine release syndrome
FFPE: Formalin fixed paraffin embedded
FL: Fetal liver
GEMM: Genetically engineered mouse model
GM-CSF: Granulocyte–macrophage colony-stimulating factor
GvHD: Graft versus host disease
HIS: Human immune system
HLA: Human leukocyte antigen
HSC: Hematopoietic stem cells
Hu-BLT: Human bone marrow, liver, thymus
Hu-CDX: Humanized CDX
Hu-PBL: Human peripheral blood lymphocytes
Hu-PDX: Humanized PDX
Hu-SRC: Human stem repopulating cells
ICI: Immune checkpoint inhibitors
IL-3: Interleukin 3
IL-6: Interleukin 6
IO: Immuno-oncology
IRAE: Immune-related adverse event
MHC: Major histocompatibility complex
MSC: Mesenchymal stem cells
NK: Natural killer
NOD: Non-obese diabetic
NSG: NOD.*Cg-Prkdc*^scid^ *Il2rg*^tm1Wjl^ /SzJ
NSG-dKO: NSG-(*K*^*b*^* D*^b^)^null^(*IA*)^null^
NSG-IL15: NSG interleukin 15
NSG-SGM3: NSG tg expression KITLG, CSF2 and IL-3
NT: Neurotoxicity
OS: Overall survival
PBMC: Peripheral blood mononuclear cells
PD-1: Programmed cell death protein 1
PD-L1: Programmed death ligand 1
PDO: Patient derived organoids
PDX: Patient derived xenograft
SCF: Skp1-Cullin-F-box protein
SPMC: Splenic mononuclear cells
TAM: Tumor associated macrophages
TCR: T-cell receptor
TIL: Tumor infiltrating lymphocyte
TLS: Tumor lysis syndrome
TME: Tumor microenvironment
UBC: Umbilical cord blood
VEGF: Vascular endothelial growth factor
